# Integrated marketing communications: a strategic priority in health and medicine

**DOI:** 10.1186/s12913-020-05606-7

**Published:** 2020-09-15

**Authors:** James K. Elrod, John L. Fortenberry

**Affiliations:** 1 Willis-Knighton Health System, 2600 Greenwood Road, Shreveport, LA 71103 USA; 2 LSU Shreveport, 1 University Place, Shreveport, LA 71115 USA

**Keywords:** Integrated marketing communications, Promotion, Advertising, Hospitals, Healthcare

## Abstract

**Background:**

Healthcare establishments portray themselves to their patient populations using many communicative mechanisms. Perhaps the first avenues that come to mind are the outward conveyances of the marketing communications mix, including advertising, personal selling, sales promotion, public relations, and direct marketing. But other prominent communicators also exist, including the people employed by healthcare institutions, the places in which services are delivered, and the brands that represent given establishments. This wide variety of communicative mechanisms is somewhat of a mixed blessing, affording myriad options, but also necessitating extra care and attention in developing associated marketing communications.

**Discussion:**

Now more than ever, health and medical establishments have at their disposal communications options capable of addressing most any conveyance want or need. The marketing communications mix, once somewhat limited due to industry tradition, is now fully accessible and widely used in the health services industry, providing immense opportunities to connect with patients. Options for signage, building designs, servicescape amenities, employee uniforms, and the like also are teeming, providing myriad avenues for making positive impressions. But burgeoning options while beneficial also intensify obligations for ensuring that marketing communications are properly integrated, with this particular article describing this imperative need and its implications for communicative success in the healthcare industry.

**Conclusions:**

By integrating marketing communications, health and medical providers are able to create synergies between and among selected conveyance mechanisms, amplifying performance and increasing the likelihood of reaching communicative goals. Achieving such cohesion requires devoted planning in an effort to coordinate verbal and visual manifestations to express desired imagery and appeals to target audiences. As extensive benefits are derived from integrated marketing communications, healthcare establishments should consider associated pursuits to be a strategic priority.

## Background

Communicating successfully with current and prospective patients represents an essential and ongoing task for health and medical establishments [1–3]. Excellence on this front is vital for many reasons, with perhaps the most notable being that effective communications inform and enlighten audiences, generating interest and attention, prerequisites for attracting patients and acquiring market share [2, 4–6]. Communications, of course, must be appealing to target markets, necessitating steps to ensure positive portrayals at every opportunity, with healthcare establishments turning to many promotional mechanisms to engage desired patient populations. In such pursuits, perhaps the first avenues that come to mind are the outward conveyances of advertising, public relations, sales promotion, and other components of the traditional marketing communications mix, with these pathways being traversed extensively by health and medical organizations in bids to connect with audiences [2, 4, 7, 8].

Indeed, the components of the marketing communications mix are tried and true methods for engaging patient populations, but they are not the only avenues available for doing so. Other prominent communicators also exist, including the people employed by healthcare institutions, the places in which services are delivered, and the brands that represent given establishments, affording extended means for informing and enlightening customer groups [2, 4, 7, 9–12]. This wide variety of communicative mechanisms, however, is somewhat of a mixed blessing. While the burgeoning array of options affords tremendous utility, healthcare providers must direct extra care and attention when assembling associated conveyances to ensure cohesion between and among promotional elements. Such cohesion yields integrated marketing communications which should be a strategic priority for health and medical establishments. This particular article shares a range of insights on integrated marketing communications, providing useful guidance for healthcare organizations seeking to bolster their patient engagement efforts.

## Discussion

Now more than ever, health and medical establishments have at their disposal communications options capable of addressing most any conveyance want or need. Today, healthcare institutions routinely make use of the full marketing communications mix, calling upon its five components of advertising, personal selling, sales promotion, public relations, and direct marketing as desired to reach their target audiences [2, 4, 13]. This starkly contrasts with prevailing healthcare industry mindsets of the not-so-distant past (ca. 1980s and earlier) which frowned on using the full power of the marketing communications mix. Notably, advertising was viewed to be beneath the dignity of medical providers and potentially detrimental to established patient referral patterns. But associated resistance ended in the 1980s, aided notably by governmental scrutiny of the American Medical Association’s ban on its members’ use of advertising, paving the way for advertising to flourish, making the marketing communications mix fully accessible and acceptable for use in the health services industry [1, 5, 7, 9].

Beyond the components of the marketing communications mix, foundational elements—people, places, and things that communicate on behalf of healthcare institutions—also present more opportunities than ever to make positive, enduring impressions. Cutting edge training programs, stylish uniforms, and the like have the potential to dramatically elevate the actions and appearances of healthcare personnel; modern exterior and interior signage options, especially digital variants, are capable of capturing attention like never before; and contemporary servicescape designs and associated amenities have the power to greatly impress and inspire target audiences. These and similar elements convey volumes of information to others, arguably projecting details as robustly as anything within the traditional marketing communications mix, crucially impacting the perspectives of current and prospective patients and presenting immense opportunities to influence associated patronage decisions [2, 4, 7, 9–12].

But burgeoning communicative options, while beneficial, also intensify obligations for ensuring that marketing communications deployed by given healthcare institutions are properly integrated to present a cohesive picture to target audiences. Integrated marketing communications (IMC) happens to be a formal term in the discipline of marketing. It is defined as “the coordination of all of the marketing communications efforts of an organization for the purpose of ensuring the consistent presentation of promotional messages to target audiences” [2], p. 286. Essentially, achieving integrated marketing communications yields a highly-desired communicative state where all conveyances telegraphed by an establishment over a given period of time are linked together. This typically is realized through the use of a defined collection of verbal and visual manifestations (e.g., logos, slogans, color schemes, themes), leading audiences to view each communication to be part of a comprehensive body of work presenting the establishment clearly and cohesively. Integration effectively creates synergies between and among all of the promotional elements deployed by an organization, amplifying the impact potential of communicative efforts [2, 4, 14–16].

In the absence of such integration, communicative elements lack effective linkages. Imagine, for example, a scenario where a healthcare establishment, on adopting a new slogan, updates its advertising to feature the new expression, but neglects to revise its other promotional materials, accordingly. Similarly, consider a situation where corporate colors have been updated, but some marketing communications continue to make use of the former color array. Given different creative treatments, communicative synergies cannot be leveraged, diminishing impact that otherwise would be possible via proper communicative integration. Indeed, the necessity for integrating marketing communications is so pronounced that entire textbooks have been written on the topic (e.g., [17–21]), demonstrating its associated value and importance.

Achieving a marketing communications presence that would be considered to be integrated requires extensive attention to detail. As various forms of promotion are developed, efforts must be directed toward ensuring that they work in harmony together, creating a cohesive, interrelated body of work [2, 4]. As content which portrays healthcare establishments (e.g., signage, advertisements, sales promotions, websites, stationery, direct mail) usually is produced over time, the need for vigilance is quite obvious, as without such, incongruence can appear in the verbal and visual artifacts representing institutions, undermining efforts to achieve and maintain integration between and among components. Such incongruence can range from subtle (e.g., an updated font for use on business cards and letterhead is introduced, but stationery featuring the old font continues to be circulated) to very obvious (e.g., a logo is revised, but signage continues to display the former identity), with such inconsistencies, regardless of magnitude, undermining communicative power and potential [4].

To foster presentation of a consistent image across all marketing communications, health and medical establishments can benefit by developing a creative style guide, a comprehensive marketing communications document which presents depictions of approved verbal and visual elements representing given institutions and supplies detailed guidelines for their use. The exact composition of such guides is dependent on the wants and needs of given healthcare establishments, but typical areas that are addressed include (1) logos, noting their proper display, use, and associated restrictions, (2) creative treatments, outlining approved typography, color schemes, and associated stylistic elements, (3) messaging characteristics, noting approved slogans, storylines, and related content, (4) audience profiles, indicating served markets, associated targets, and demographic attributes, (5) communication avenues, identifying components of the marketing communications mix and any supplementary communications pathways that are to be pursued, and (6) authorization processes, outlining protocols for presenting assembled communications to institutional leaders for review and approval, permitting dissemination.

The activity of developing these guides, in and of itself, is helpful in that it forces institutional leaders to think deeply on the verbal and visual manifestations which represent their given organizations, providing an opportunity to examine and potentially improve methods and manners of presenting their establishments to target audiences. This also aids in achieving communicative cohesion, as components are developed in an orderly fashion, something which avoids patchwork assembly methods which inadvertently can foster incongruity [2, 4, 10, 22]. The all-important authorization process is also addressed, further ensuring compliance with designated standards. By adhering to defined creative protocols, a clear identity emerges, essentially yielding consistent creative signatures which amplify all marketing communications forwarded on behalf of healthcare institutions.

**Fig. 1:**
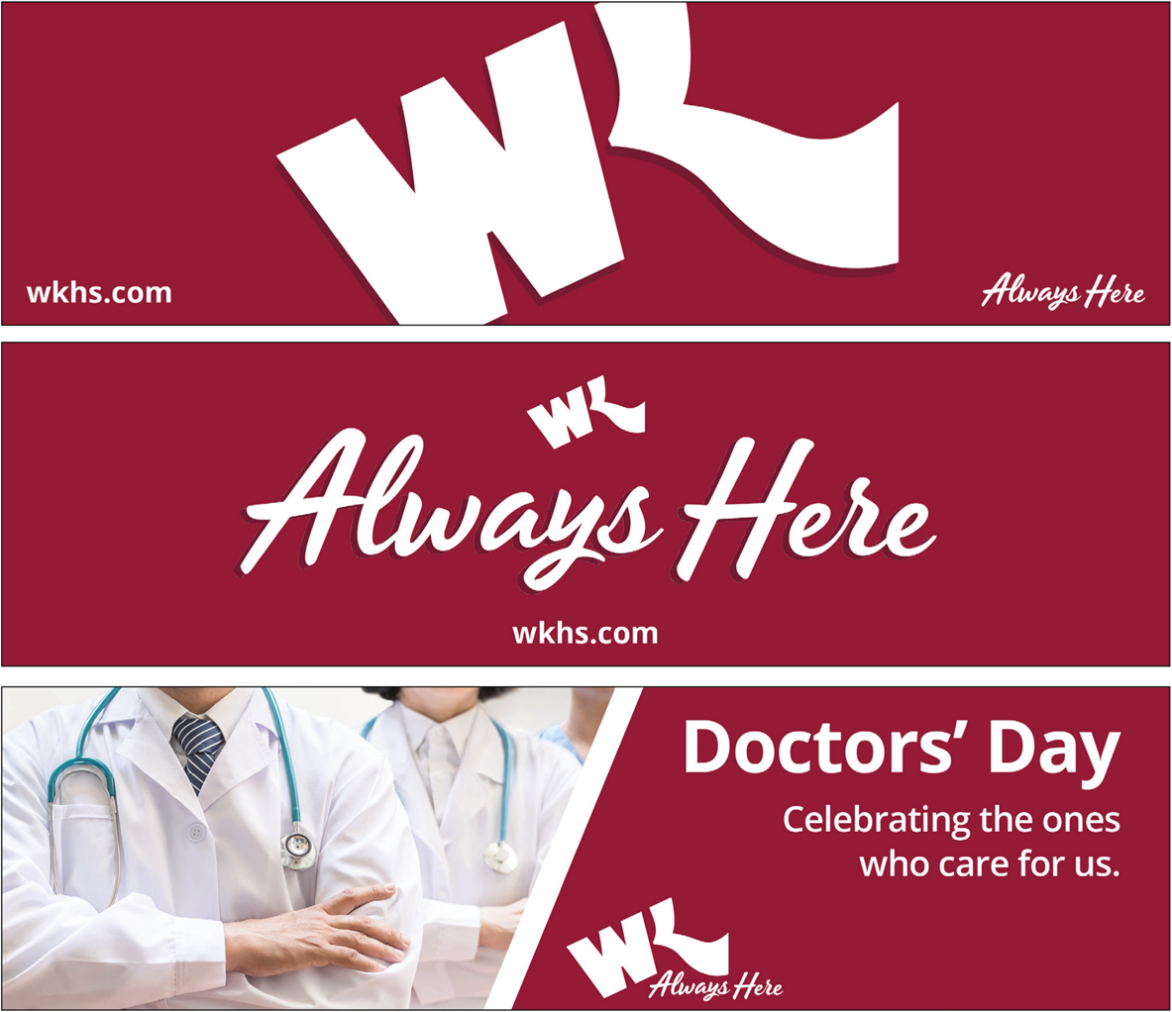
Several recent billboard advertisements promoting Willis-Knighton Health System

**Fig. 2:**
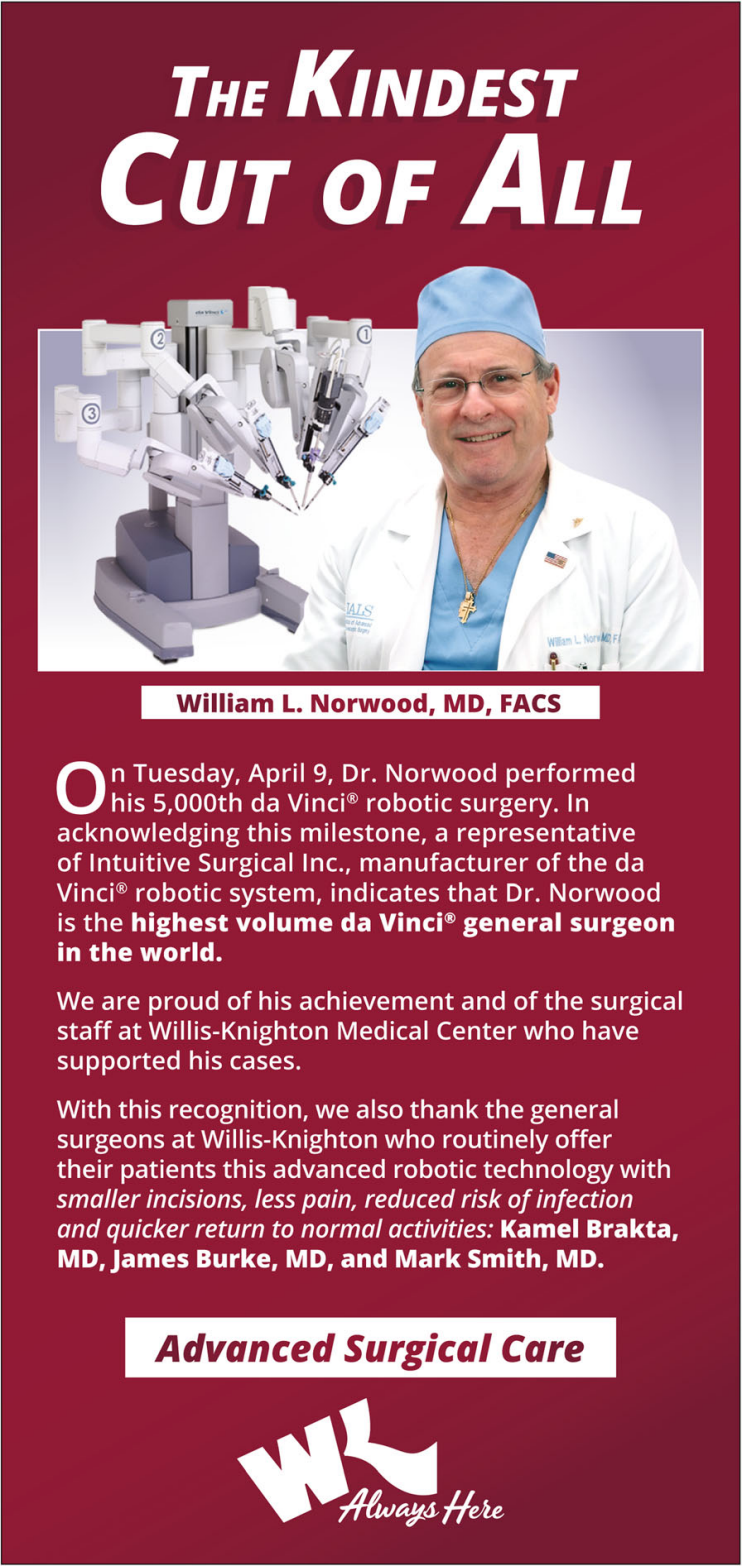
A recent newspaper advertisement promoting Willis-Knighton Health System

In the 1970s, a particularly formative time period for the institution, Willis-Knighton Health System laid the groundwork for enduring integrated marketing communications by focusing on creative treatments and applications, with one of the most essential being the development of an appealing logo. Today, this logo is one of the most widely recognized in the marketplace and it anchors virtually every communication forwarded by the institution [9, 10]. Coupled with consistent color schemes, slogans, and manners and methods of presentation, Willis-Knighton Health System’s particular creative treatment, regardless of the service line to which it is applied, builds communicative bridges between and among each and every conveyance, naturally supplying the critical linkages that afford integrated marketing communications, as the sample billboard and newspaper advertisements, provided in Figs. 1 and 2, and the sample television commercials, available at https://www.wkhs.com/video/commercials, illustrate.

Applications of Willis-Knighton Health System’s creative style are not limited to conveyances of the traditional marketing communications mix; they influence decisions pertaining to anything which communicates on behalf of the institution, guiding signage decisions, employee uniform designs, and so on, yielding a universal creative portrayal of the institution to its audiences. Such results stem from the devoted use of a creative style guide which, once assembled and approved, directs the development and dissemination of content, providing reasonable assurances that presentations and portrayals of given institutions will be consistent.

A healthcare establishment’s achievement of integrated marketing communications is best ascertained by viewing the totality of communicative elements deployed by the given institution. Interior and exterior signage, stationery, employee uniforms, promotional brochures, advertisements, direct mail pieces, website designs and associated promotional content, and more should be examined comprehensively. If each element when viewed individually appears to be part of the larger body of work, then integration pursuits can be deemed to have been successful. If not, corrective actions need to be taken to bring noncompliant promotional elements into compliance with the communicative aims expressed in the associated creative style guide, affording harmony between and among all conveyances.

While the concept of integrated marketing communications might seem so obvious as to not warrant discussion, environmental complexities, propensities to lose sight of the bigger picture, routine distractions, and the like have the potential to undermine and erode efforts to ensure cohesion between and among communicative components. As noted earlier, if proper attention is not directed toward the assembly and management of elements promoting healthcare establishments, incongruence eventually will emerge, diminishing the communicative power and potential of given expressions. The introduction and use of creative style guides, coupled with ongoing discussions regarding the importance of integrated marketing communications, will help healthcare establishments stay on course for achieving harmony across all communications, reaping associated benefits.

## Conclusions

By integrating marketing communications, health and medical providers are able to portray themselves clearly and consistently to their audiences, something that is absolutely essential for the best communicative outcomes. Further, synergies will emerge between and among the deployed conveyance mechanisms, amplifying performance and increasing the likelihood of reaching communicative goals, ultimately improving the return on investment generated by the associated marketing communications. Achieving such cohesion requires devoted planning in an effort to coordinate verbal and visual manifestations to express desired imagery and appeals to target audiences, with guiding protocols and reminders to maintain integration being essential as a defense against oversights and distractions which could lead to communications incongruence. As extensive benefits are derived from integrated marketing communications, healthcare establishments should consider associated pursuits to be a strategic priority.

## Data Availability

Not applicable.
